# Sugarcane Bagasse as the Source of Nanocrystalline Cellulose for Gelatin-Free Capsule Shell

**DOI:** 10.1155/2022/9889127

**Published:** 2022-02-14

**Authors:** Zakir Sabara, Alfirah Mutmainnah, Ummu Kalsum, Irma Nur Afiah, Ismalia Husna, Antomi Saregar, Irzaman, Rofiqul Umam

**Affiliations:** ^1^Faculty of Industrial Technology, Universitas Muslim Indonesia, Makassar, Indonesia; ^2^Department of Parasitology, Faculty of Medicine, Universitas Malahayati, Bandar Lampung, Indonesia; ^3^Department of Physics Education, UIN Raden Intan Lampung, Bandar Lampung, Indonesia; ^4^Department of Physics, Bogor Agricultural University, Bogor, Indonesia; ^5^Department of Applied Chemistry for Environment, Kwansei Gakuin University, Sanda, Japan

## Abstract

Gelatin-free capsules are possibly produced through an innovation which involves utilizing environmentally friendly materials derived from plants such as bagasse which are produced into nanocrystalline cellulose (NCC). This research was conducted to report the extraction and characterization of NCC from the abundant industrial plantation waste of sugarcane and its application as the base material for gelatin-free capsule shell material. The process involved using different concentrations of NCC at 1%, 2%, 4%, and 7% (in wt. %) with the addition of 1% hydroxypropyl methylcellulose (HPMC) (in wt. %) and 1% carbopol (in wt. %). Moreover, the NCC capsules obtained from sugarcane bagasse were tested for moisture content, tensile strength, elongation, solubility, and pH. The results showed that sugarcane bagasse contains 40–50% cellulose, 6.15%–9.5% moisture content which indicates they are potentially better in terms of storage, 7.25–7.85 pH, and 0.05–0.136 MPa gel strength, and the elongation value ranges from 7.19 to 87.51%. These values were discovered to have satisfied the standard requirements as indicated by the optimal concentration of 4% NCC +1% HPMC, which is in line with the Japanese Industrial Standard (JIS), thereby leading to the consideration of the material safe to be used as raw material in making capsule shells.

## 1. Introduction

Indonesia is a country with a tropical climate which allows different types of plants such as sugarcane (*Saccharum officinarum* L.) to thrive. This plant is normally used as the raw material to produce sugar to meet the needs of the community. It is important to note that the production process usually has 90% bagasse, 5% molasses, and 5% water with the bagasse reported to be used only as a fuel for sugarcane processing [1]. Meanwhile, the ash from the uncontrolled burning of bagasse has been proven to be causing serious air pollution problems [2], thereby [3] making it necessary to find better environmentally friendly alternatives [4]. This led to the selection of sugarcane as the raw material to be used in producing nanocrystalline cellulose (NCC) for gelatin-free capsule shell material due to its several advantages such as the 40–50% cellulose content [5] and availability in large quantities.

Capsules are one of the pharmaceutical dosage forms normally used to package drugs in powder form to cover the taste and smell considered by some people to be irritating, thereby making the treatment process easier [6]. It was reported that several types of capsules especially those containing gelatin usually have the problem of stability [7]. This is associated with the fact that gelatin is one of the ingredients in animal protein mostly obtained from the bones and skins of livestock such as cows and pigs from slaughterhouses by capsule manufacturing factories [8]. It is also important to note that gelatin has also been applied in the food industry in addition to medicine and pharmaceuticals due to its ability to form hydrogels in the pH range without the aid of ionic or other additives [9]. However, gelatin capsules cannot be used for drugs and highly hygroscopic drugs due to the fact they also have high moisture content and need to be stored at 45–60% humidity. They also have the ability to experience cross-linking which can make their shell very hard [10]. These shortcomings led to the production of capsules from nongelatin materials [7].

Capsule shells have been manufactured using hydroxypropyl methylcellulose (HPMC) [11] which has better physical stability when compared to gelatin. This HPMC, however, has a lower tensile strength value due to the lack of NCC, and this normally causes problems during the capsule shell manufacturing process [12].

This study was used to manufacture capsule shells using bagasse as the raw material for the NCC while HPMC and carbopol were used as the base formula for the mixture. Carbopol is a fine white, acidic, and hygroscopic powder selected due to its ability to easily disperse in water and function as a gel base with sufficient viscosity in small concentrations, thereby leading to small dispersion and long adhesion. The main goal of this study is to produce gelatin-free capsule shells with a higher tensile strength value than those produced in previous studies [13]. It was also focused on measuring certain parameters such as moisture content, solubility, pH, and disintegration time as well as mechanical strength such as tensile strength and elongation to determine their conformity with the Japanese Industrial Standard (JIS) [14].

## 2. Materials and Methods

### 2.1. Sugarcane Waste Materials and Preparation

The bagasse used as the raw material was obtained from the industrial waste of sugar cane plantations in Takalar Regency, South Sulawesi, Indonesia. In the process, the bagasse is washed first to remove impurities such as soil and others. Furthermore, the process of drying the bagasse is carried out using sunlight for 1-2 days depending on the weather. After the drying process is complete, the bagasse is then cut and ground using a milling machine (FOMAC FCT-Z500) to form a powder. Based on the information obtained from the performance specifications of the milling machine (FOMAC FCT-Z500), it can produce powders with a fineness level reaching 20–180 mesh with an engine rotation speed of 2800 rpm. These processes were conducted between July and September 2021 at the Chemical Engineering Research Laboratory, Faculty of Industrial Technology, Indonesian Muslim University, Makassar, South Sulawesi, Indonesia.

### 2.2. Cellulose Extraction (CE)

A total of 50 grams of bagasse powder was hydrolyzed using 400 ml of 3.5% HNO_3_ and heated at 90°C for 2 hours. After that, it was washed, filtered, bleached with 3.5% NaOCl, and boiled for 10 minutes before it was finally washed and filtered again. This was followed by the CE which involved adding NaOH to 17.5% of the total bagasse powder and heating at a temperature of 80 °C for 30 minutes, and then, the washing and filtering processes were conducted again, followed by bleaching with a 3.5% NaOCl solution at a temperature of 100°C for 10 minutes [15]. The final result of this bleaching process was washed up to the moment the pH became neutral and later dried in an oven at 60°C to obtain alpha-cellulose which was characterized using FTIR (Fourier-transform infrared spectroscopy) to analyze its functional groups.

Although the bagasse has been processed to form a powder, in the next process, which is hydrolysis, it is necessary to add 500 ml of 50% sulfuric acid until the hydrolysis process is stopped by adding distilled water so that the final sample obtained is in liquid form. The IR spectrometry technique used in this study was transmittance by adding KBr as a crystalline. The transmission method is one of the techniques for measuring absorption/molecular vibration with an FTIR. The sample is subjected to a direct laser beam to determine the vibrational pattern of the molecule. In this method, solid samples are made in the form of clear pellets with the help of KBr. The KBr sample pellets were then analyzed using an FTIR. The measurements were made in the range of wavenumbers from 4000 to 400 cm^−1^.

### 2.3. Nanocrystalline Cellulose (NCC) Synthesis

**Figure 1:**
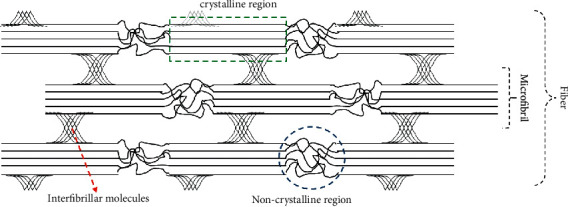
The crystalline and amorphous structures of cellulose. The red dotted arrow is the degradation of the amorphous or noncrystalline region with the weakest bond, this location being the site of the production of nanocrystalline cellulose or interfibrillar molecules.

Nanocrystalline cellulose (NCC) was prepared by acid hydrolysis using sulfuric acid solution with 50% concentration used to hydrolyze sugarcane bagasse cellulose for 30 minutes at 45°C. It is important to note that cellulose fibers degrade [16]when they are reacted with acids, and this was observed to have started at the region of the weakest bond which is the amorphous or noncrystalline region, as indicated in Figure 1. The reduced amorphous region caused the crystallinity of cellulose to increase, thereby leading to nanocrystalline cellulose or interfibrillar molecules.

The sugarcane bagasse powder dried through cellulose extraction was also hydrolyzed with 500 ml of 50% sulfuric acid at 45°C for 30 minutes, and the process was stopped by adding distilled water to the reaction mixture. The colloid produced was centrifuged and later dialyzed with distilled water to have a pH of 6. From this process, NNC was obtained and characterized again through FTIR to determine its functional group.

### 2.4. Capsule Casting

The gel was produced by dissolving NCC at 1%, 2%, 4%, and 7% (in wt. %) and then the mixing process was conducted with the addition of 1% HPMC (in wt. %) and 1% carbopol (in wt. %) each into distilled water at a temperature of 30°C for 30 minutes and a speed of 500 rpm. The gel formed was printed with a capsule printer, and the gel film attached to the capsule printer was dried in an oven at 60°C for 3 hours, and then, the capsule was removed from the mold and its properties including moisture content, tensile strength, elongation, solubility, pH, and the time evaluation of capsule shell rupture were analyzed.

On the other hand, PEG-400 and glycerin are added to the capsule shell formulation as plasticizers. The addition of a plasticizer reduces the stiffness of the polymer and increases the flexibility of the resulting capsule shell [17]. PEG-400 was chosen in the formulation because it can absorb moisture from the capsule shell. The concentration of PEG-400 used was 2%.

### 2.5. Disintegration Test

The disintegration time test used a tool known as the disintegration test. In its application, the disintegration time of the capsule shell is very important to estimate when the drug in the capsule will start to work on the human body [18]. The process of testing the disintegration time was carried out by inserting 1 capsule and water at 37°C into each glass tube. The glass tube containing the capsule is then placed into the basket of the disintegration tester machine [19]. After the engine is started, the basket moves up and down in the transparent solution at a speed of 29–32 rpm. The disintegration time interval is 5–15 minutes. The capsules within the time limit stated in each monograph were observed until the capsule shell disintegrates or dissolves in water [20].

## 3. Results and Discussion

### 3.1. Proximate and Ultimate Analyses of the Sugarcane Bagasse

The proximate and ultimate analyses were conducted to determine the quality of the starting material which is the sugarcane bagasse used for the extraction of cellulose and NCC. The moisture content, hydrogen, oxygen, and ash of the raw materials were observed to have a significant effect on the properties of the NCC produced [17] as indicated in the proximate results presented in Table 1. It was discovered that the composition of the bagasse produced is quite different from the previous report provided by Adebisi et al. [1]due to the use of different sugarcane species, as reported to have used *Saccharum officinarum* which originates from Nigeria, Africa.

The sugarcane bagasse used in this study had an ash content value of 2.5%, and this is considered to be a good quality raw material to manufacture soft capsule shells due to the fact that it is more than 0.5%. However, the hydrogen and oxygen contents were fairly high at 6.5% and 44%, respectively, thereby affecting the dispersion properties in water [21]. This led to the addition of a mixture of HPMC and carbopol to improve the quality of the capsule shell produced to ensure that it is easily dispersed in water and also produced in small concentrations to serve as a gel base with sufficient viscosity to increase the adhesion [11].

### 3.2. Analysis of Cellulose Extraction (CE) and Synthesis of Nanocrystalline Cellulose (NCC)

**Figure 2:**
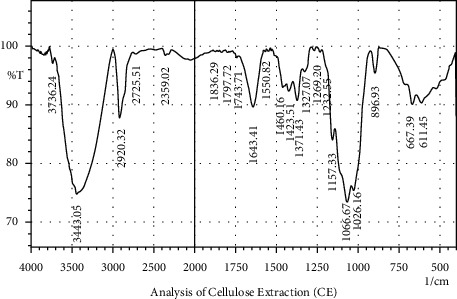
The results of the analysis of cellulose extraction (CE) using FTIR.

**Figure 3:**
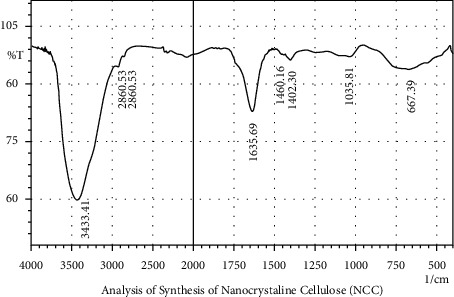
The results of the analysis of nanocrystalline cellulose (NCC) using FTIR.

Bagasse prepared into powder before the CE and NCC processes was analyzed using FTIR to determine its functional groups or contents such as cellulose, hemicellulose, lignin, minerals, wax, and other components [1]which have the ability to affect its properties and characteristics as a base material for capsule shells. The results of the CE and NCC characterization analysis are shown in Figures 2 and 3 and described in Table 2.

The FTIR spectra for the CE and NCC presented in Figures 2 and 3showed there is a band part of the CE not found in the NCC while a difference was observed in the 896.93 cm^−1^ recorded in Figures 2 and 1,157.33 cm^−1^ in Figure 3. Moreover, the FTIR characterization showed there were changes after the bagasse had been processed into cellulose and nanocrystalline cellulose, while several bands were observed to be shared by all samples (CE and NCC) analyzed, as shown in the areas of 3,443.05 cm^−1^ and 3,433.41 cm^−1^ which indicate the moisture content of the O-H stretching vibration as well as areas of 2,920.32 cm^−1^ and 2,860.53 cm^−1^ which show the presence of carbon and hydrogen bonds in Sp3 C-H stretching [22]. Furthermore, the peaks in the area of 1,371.43 cm^−1^ and 1,403.30 cm^−1^ indicate the presence of cellulose and lignin in the C-O or C-H bonds, while those of 1,643.41 cm^−1^ and 1,635.69 cm^−1^show that water was absorbed by cellulose. All these results are presented in Table 2.

### 3.3. Physicochemical Properties of Cellulose Extraction (CE) and Synthesis of Nanocrystalline Cellulose (NCC)

The physicochemical properties of sugarcane bagasse presented in Table 3 showed that the cellulose extracted ranged between 40 and 50%. It is important to note that the cellulose content can be influenced by several factors such as the type of sugarcane, the bleaching process [23], and the extraction temperature [24]. This is indicated by the fact that the yield produced in this study was higher than those from previous studies and other types of plants [1]. Furthermore, the degradation and hydrolysis processes were conducted to synthesize nanocrystalline cellulose (NCC) as the basic material to produce capsule shells at different concentrations at 1%, 2%, 4%, and 7%, with the addition of hydroxypropyl methylcellulose (HPMC) and carbopol.

**Figure 4:**
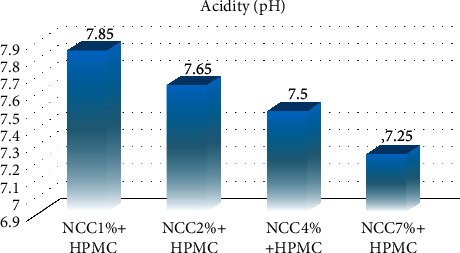
The pH value for 1%, 2%, 4%, and 7% NCC with hydroxypropyl methylcellulose (HPMC).

pH is an important factor to determine the quality of nanocrystalline cellulose (NCC) and ensure it is safe to use as a raw material. It was discovered that the pH values of the NCC ranged from 7.25 to 7.85, as shown in Figure 4, and this means it complies with the Japanese Industrial Standard (JIS) for capsule shells, which are estimated at 6.5–8.5. This further showed that the material is safe to be used as raw material to produce capsule shells [25].

**Figure 5:**
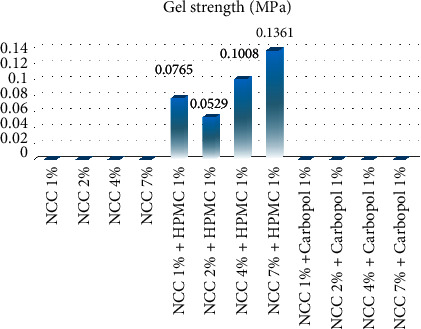
The gel strength (MPa) values for 1%, 2%, 4%, and 7% NCC with hydroxypropyl methylcellulose (HPMC) and carbopol

Gel strength is the most important physical property of NCC due to its ability to describe the strength of intermolecular cohesion [12] and proportionality to its molecular weight [11]. The results presented in Tables 3 and 4 and Figure 5 showed that NCC 7% with HPMC has the highest gel strength of 0.1361 MPa, while NCC 2% with HPMC has the lowest gel strength of 0.0765 MPa. These values were observed not to be in line with the Japanese Industrial Standard (JIS) [26], which requires a minimum of 0.3 MPa for NCC. In a previous study, Nurilmala et al. explained that gel strength was influenced by the presence of gelatin and was determined by the viscosity value of each gelatin [27]. In this study, the low value of gel strength was estimated due to the absence of gelatin in the bagasse extract. However, the gelatin-free capsule shell has the advantage that it can be used by someone who is allergic to gelatin.

**Figure 6:**
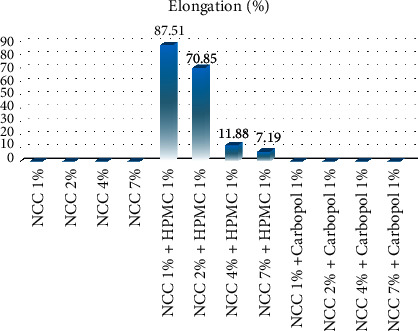
The elongation (%) values for 1%, 2%, 4%, and 7% NCC with hydroxypropyl methylcellulose (HPMC) and carbopol.

The elongation value was observed to have met the Japanese Industrial Standard (JIS) [14]which is required to be at a minimum elongation of 5% as indicated in Figure 6. This is due to the influence of the addition of glycerol as a plasticizer. The volume of glycerol being too high causes the distribution of the constituent components to be uneven [28]. This indicates more NCC content has the ability to increase the hydrogen bonds formed between the same polymers, thereby requiring more energy to break the bonds but having the ability to break them at low strains [27]. Moreover, the other NCC formulas have lower tensile strength values due to fewer hydrogen bonds, and this leads to weaker properties, easy deformation, and breakage at higher strains [7]. This was observed to be in line with the findings of Djafari Petroudy that the addition of more components of bagasse increased the tensile strength but reduced the elongation value [29]. The addition of HPMC was also observed to have increased the mechanical strength of the film.

**Figure 7:**
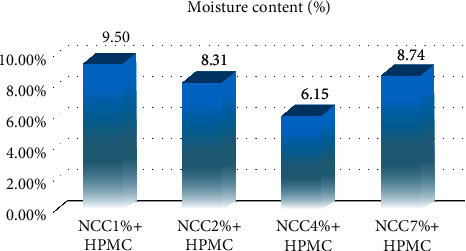
The moisture content (%) value for 1%, 2%, 4%, and 7% NCC with hydroxypropyl methylcellulose (HPMC).

The moisture content of all variations of NCC concentration was analyzed to determine the water content or humidity of the capsules [5], which is related to the resistance of the capsule shell to microbial activity, especially bacteria [30]. The moisture content required by the Japanese Industrial Standard (JIS) is <10% [14], but those recorded in this study ranged from 6.15% to 9.5% as indicated in Figure 7 which is lower than the 12.5–15% in commercial capsule shells. This means all the variations of NCC have the required water content and have the potential to be better in terms of storage as containers for drugs are considered to be sensitive to moisture and are not easily overgrown with microbes, thereby extending the shelf life of the capsule [31].

### 3.4. Capsule Shell

**Figure 8:**
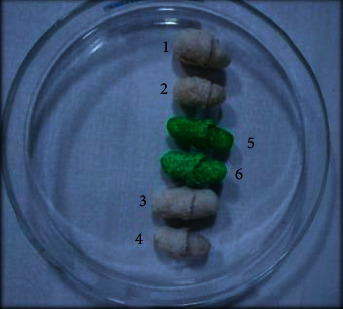
Capsule shells produced from the NCC of sugarcane bagasse waste mixed with hydroxypropyl methylcellulose (HPMC) and carbopol. (1) Capsules were produced at 1% NCC concentration +1% HPMC; (2) capsules were produced at 2% NCC concentration +1% HPMC; (3) capsules were produced at 4% NCC concentration +1% HPMC; (4) capsules were produced at 7% NCC concentration +1% HPMC; (5) capsules were produced at 4% NCC concentration +1% carbopol; and (6) capsules were produced at 7% NCC concentration +1% carbopol.

Nanocrystalline cellulose (NCC) capsules were produced at 1%, 2%, 4%, and 7% concentration. It is important to note that the capsule forming device affected the shape of the capsule, as indicated in Figure 8. The preliminary formulation of the NCC concentration was in the form of a film, but the results showed that the film was not formed but rather returned to form a colloid because the viscosity was too low. This led to the addition of HPMC to increase the flexibility and allow it to be printed on the capsule printer [21]. The HPMC additives were selected due to their proven compatibility with NCC [32].

The HPMC concentration used was 1%, and this allowed 1%, 2%, 4%, and 7% NCC concentrations to be printed on the capsule printer. It is also important to note that 1% carbopol was also added, and this material was selected due to its easy dispersion in water at a small concentration of 0.05–2%, but it has a very high viscosity which limits its ability to form a film. PEG-400 and glycerin were also added as plasticizers to reduce the stiffness of the polymer and increase the flexibility of the capsule shell produced [17]. The decrease in the polymer strength was caused by the reduction in the internal hydrogen bonds of the intermolecular bonds [33]. Meanwhile, the PEG-400 was selected due to its ability to absorb moisture from the capsule shell [10] and was used at a 2% concentration, which is within the <30% allowed for parenteral preparations.

The solubility of nanocrystalline cellulose (NCC) was tested using three solvents, which included distilled water, acetone, and n-hexane at different polarity levels of polar, semipolar, and nonpolar. The results showed that the NCC can be dispersed in distilled water but not in acetone and n-hexane. This is in accordance with the Japanese Industrial Standard (JIS) [14] that NCC is easily dispersed in water and forms a colloidal solution but is insoluble in acetone and n-hexane.

### 3.5. Capsule Disintegration Test

The capsule disintegration was tested to determine the time for the capsules to disintegrate, and the results are presented in Table 5. This is necessary because the capsule needs to decompose within a certain period for the ingredients encapsulated to be absorbed by the body [10–12]. The test was conducted on Milli-Q water medium at a temperature of 37°C ± 2°C, which is adapted to human body temperature and a total of 6 capsule shells were used [8] at 1%, 2%, 4%, and 7% NCC with 1% HPMC. The results showed the capsule shell required 30 minutes to disintegrate.

Research on making capsules using bagasse which is synthesized into a form of nanocrystalline cellulose (NCC) was analyzed with several test parameters to obtain the optimal concentration of NCC in the manufacture of capsules. The values of gel strength (Table 4), elongation (Figure 7), and disintegration test results (Table 5) show that the higher the concentration of NCC with the addition of HPMC, the higher the values of gel strength and disintegration tests will be, but higher NCC concentrations have lower elongation values. The gel strength is lower than the Japanese Industrial Standard (JIS) [14] because bagasse is a gelatin-free capsule shell material.

## 4. Conclusion

The sample with the lowest water content is 4% NCC, and this indicates it has better potential to serve as a drug container due to the fact that it cannot be easily overgrown by microbes, thereby prolonging the shelf life of the capsule. Moreover, the sample with the highest tensile strength is 7% NCC at 0.1361 MPa, but the value is below the 0.3 MPa required by the Japanese industrial standard (JIS), while the highest elongation of 87.51% was recorded in 1% NCC, which is significantly higher than the required minimum of 5%. The pH for all the variations was discovered to be between 6.5 and 8.5, and this implies the NCC is safe to use as raw material to produce capsule shells. Therefore, the optimum concentration of nanocrystalline cellulose (NCC) to manufacture capsules is 4% in combination with HPMC.

## Figures and Tables

**Table 1:** Proximate and ultimate analyses of sugarcane bagasse waste materials.

	Parameter (% w/w)		
	This work		*Saccharum officinarum*
	Average (%)	Standard deviation	Average (%) [1]
*Proximate analysis*			
Moisture	7.82	0.04	8.37
Ash	2.50	0.05	1.73

*Ultimate analysis*			
Hydrogen	6.50	0.08	3.0
Oxygen	44	0.06	22.80

**Table 2:** Functional group analysis of the CE and NCC.

Wavenumber (cm^−1^)			
CE	NCC	Range (cm^−1^)	Functional group interpretation
3,443.05	3,433.41	34,00–3,200	O-H stretching vibration
2,920.32	2,860.53	3,000–2,850	Sp3 C-H stretching
—	—	1,750–1,735	C=O stretching
1,643.41	1,635.69	1,650–1,630	OH (air)
—	—	Around 1,600 and 1,475	C=C aromatic ring
—	—	1,600–1,500	C=C aromatic ring
1,371.43	1,402.3	1,440–1,000	C-O-H bending
1,157.33	—	1,300–1,000	C-O stretching vibration
1,066.67	1,035.81	1,300–1,000	C-O stretching vibration
896.93	—	Around 850	Asymmetric C-O-C stretching vibration
—	—	900–690	=C-H stretching aromatic ring

**Table 3:** Physicochemical properties of nanocrystalline cellulose (NCC).

Properties	Sugarcane bagasse	
	NCC (1%, 2%, 4%, and 7%)	Japanese Industrial Standard (JIS-K2530) [14]
Moisture (%)	6.15–9.50%	Max 15%
Ash (%)	2.50%	Max 5%
pH value	7.25–7.85	6.5–8.5
Gel strength (MPa)	0.0529–0.1361	Min 0.3
Elongation (%)	7.5–7.85%	Min 5%

**Table 4:** Gel strength results of the capsule shells from NCC produced from sugarcane bagasse waste added with hydroxypropyl methylcellulose (HPMC).

Variation of NCC capsules with 1% HPMC	Gel strength (MPa)		Elongation (%)		Moisture content (%)	
	Average	Standard deviation	Average	Standard deviation	Average	Standard deviation
NCC 1%	0.0765	0.0003	87.510	2.602	9.500	0.470
NCC 2%	0.0529	0.0003	70.850	2.224	8.310	0.520
NCC 4%	0.1008	0.0002	11.880	2.067	6.150	0.679
NCC 7%	0.1361	0.0001	7.190	0.430	8.740	0.580

**Table 5:** Disintegration test results of the capsule shells from NCC produced from sugarcane bagasse waste added with hydroxypropyl methylcellulose (HPMC) and carbopol.

Variation of NCC capsules with HPMC	Disintegration time (minutes)						Average time (minutes)	Standard deviation
	S1	S2	S3	S4	S5	S6		
NCC 1%	7.32	5.53	5.57	8.12	6.18	8.24	6.82	1.23
NCC 2%	3.00	4.21	4.33	3.55	2.14	4.46	3.61	0.91
NCC 4%	4.30	4.24	3.54	10.23	4.08	3.46	4.97	2.59
NCC 7%	9.27	8.56	7.59	10.23	8.23	10.36	9.03	1.09

S = sample.

## Data Availability

The data used to support the findings of this study are available from the corresponding author upon request.
